# Disparities in Temporal and Geographic Patterns of Myocardial Infarction Hospitalization Risks in Florida

**DOI:** 10.3390/ijerph16234734

**Published:** 2019-11-27

**Authors:** Evah W. Odoi, Nicholas Nagle, Chris DuClos, Kristina W. Kintziger

**Affiliations:** 1 Comparative and Experimental Medicine, College of Veterinary Medicine, The University of Tennessee, Knoxville, TN 37996, USA; ewangui@utk.edu; 2 Department of Geography, The University of Tennessee, Knoxville, TN 37996, USA; nnagle@utk.edu; 3 Environmental Public Health Tracking, Division of Community Health Promotion, Florida Department of Health, Tallahassee, FL 32399, USA; Chris.Duclos@flhealth.gov; 4 Department of Public Health, The University of Tennessee, Knoxville, TN 37996, USA

**Keywords:** myocardial Infarction, hospitalization risks, geographic disparities, temporal patterns, Kulldorff and Tango’s flexible spatial scan statistics

## Abstract

Knowledge of geographical disparities in myocardial infarction (MI) is critical for guiding health planning and resource allocation. The objectives of this study were to identify geographic disparities in MI hospitalization risks in Florida and assess temporal changes in these disparities between 2005 and 2014. This study used retrospective data on MI hospitalizations that occurred among Florida residents between 2005 and 2014. We identified spatial clusters of hospitalization risks using Kulldorff’s circular and Tango’s flexible spatial scan statistics. Counties with persistently high or low MI hospitalization risks were identified. There was a 20% decline in hospitalization risks during the study period. However, we found persistent clustering of high risks in the Big Bend region, South Central and southeast Florida, and persistent clustering of low risks primarily in the South. Risks decreased by 7%–21% in high-risk clusters and by 9%–28% in low-risk clusters. The risk decreased in the high-risk cluster in the southeast but increased in the Big Bend area during the last four years of the study. Overall, risks in low-risk clusters were ahead those for high-risk clusters by at least 10 years. Despite MI risk declining over the study period, disparities in MI risks persist. Eliminating/reducing those disparities will require prioritizing high-risk clusters for interventions.

## 1. Introduction

Preventive and control strategies for acute myocardial infarction (MI), or heart attack, have resulted in substantial reductions in the incidence and overall burden of MI hospitalizations in the U.S. [1,2]. However, MI remains a leading cause of hospital admissions in the U.S. [3], accounting for 608,800 hospital discharges/stays in 2014. [4] Moreover, the burden is projected to get worse as major MI risk factors such as diabetes mellitus (DM), obesity and population aging [5,6,7] become increasingly prevalent in the future [8], making MI prevention a continuing public health priority.

Despite the overall decline in the burden of MI hospitalizations in the U.S. over time, the rates have been shown to vary widely across the country. For instance, county-level MI hospitalization rates for individuals aged 35 years and older decreased by at least 20% for 19 out of 20 states in the Centers for Disease Control and Prevention (CDC) Tracking Network between 2000 and 2008. However, the rates were higher in states in Northeast/Mid-Atlantic corridor and lower in the Mountain and Pacific states [9]. In that study, Florida stood out as the only state where the overall MI hospitalization rates increased during the study period.

Extant literature shows that cardiovascular events tend to cluster persistently in certain populations defined by geography or demographic characteristics [10,11,12,13,14]. Additionally, the burden of major cardiovascular disease (CVD) risk factors, such as obesity, diabetes, and smoking are disproportionately higher in certain geographic regions, particularly rural areas in the Southern and Southeastern U.S. states [15,16,17]. Despite the additional burden of risk in rural areas, primary and secondary preventive strategies for MI, such as public smoking bans, regulations around the sale and marketing of tobacco products and coronary revascularization, disproportionately benefit urban areas [18,19,20]. In light of these differences, it is plausible that some parts of Florida may have a disproportionately high burden of MI. Identifying these areas would enable targeting of intervention strategies to the most affected populations, leading to the improved health of all groups and reduced health disparities, which are the overarching goals of the Healthy People 2020 national public health agenda [21]. Monitoring trends in MI risks can provide key insights into the effectiveness of prevention efforts. Moreover, MI overlaps geographically with other cardiovascular diseases, such as stroke, and associated factors, e.g., hypertension, DM, obesity, etc. [13,22,23]. Therefore, our approach is useful for pinpointing regions in need of targeted interventions that reduce the burdens of MI and other chronic diseases contributing to the large and growing geographic disparities in life expectancy in Florida [24,25]. Therefore, our objectives were to: (a) investigate the spatial distribution and clusters of MI hospitalization risks, and (b) monitor trends/changes in geographic disparities in MI hospitalization risks in Florida from 2005 to 2014.

## 2. Materials and Methods

### 2.1. Study Population and Design

This retrospective ecological study used MI inpatient hospital admissions data for in Florida for the period between 1 January 2005 and 31 December 2014. The study population included all Florida residents with a primary discharge diagnosis of acute MI based on the International Classification of Diseases, Ninth Revision, Clinical Modification: ICD-9-CM diagnostic code 410.

### 2.2. Data Sources and Data Preparation

#### 2.2.1. Hospital Discharge Data

We obtained individual-level MI hospitalization data collected by the Florida Agency for Health Care Administration (AHCA) from the Florida Department of Health (DOH). The AHCA data include discharge claims from all Florida hospitals except Veterans Affairs, Indian Health Services, and prison or state-owned facilities; hence, it represents surveillance with 100% coverage for noninstitutionalized hospitals. We extracted the following variables: admission date; primary diagnosis; patient age, sex and race; and zip code and county of residence.

#### 2.2.2. Population Data

We obtained annual county-level population estimates for sex and age categories matching hospitalization data (i.e., 0–34, 35–44, 45–54, 55–64 and ≥65 year-olds) from the DOH [26] and then used them as denominators for calculating sex- and age-specific annual MI hospitalization risks. Although the 2000 U.S. standard population is recommended for age-adjustment of age-dependent health events [27], the 2010 U.S. standard population reflects the most recent actual age compositions of the U.S. population, and it also falls within the range of our data collection. Moreover, since the risk of MI increases with age, using a younger population with a lower proportion of older ages could yield lower age-adjusted risks. Therefore, we used the decennial data for the 2010 U.S. population from American FactFinder website [28] for direct age adjustment, as it may provide us with more realistic and more current risk estimates [29], and compared this to the age-adjusted rates using the widely accepted 2000 U.S. standard population.

#### 2.2.3. Cartographic Boundary Files

County-level base maps used for mapping were downloaded from the U.S. Census Bureau website [30].

### 2.3. Descriptive Statistics

The County was the spatial unit of analysis. We aggregated the MI data for each county by sex and age (i.e., 0–34, 35–44, 45–54, 55–64 and 65 years and older) by 2-year increments. We then used these counts along with county population estimates and both the 2000 and 2010 U.S. standard populations to calculate sex- and age standardized (per 10,000 population) MI hospitalization risks [27]. We also stratified state-level MI hospitalization data at the beginning (2005–2006) and end of the study (2013–2014) by sex, race (Hispanic white, non-Hispanic Black, Other), and ethnicity (Hispanic, non-Hispanic) and age-standardized them to both the 2000 and the 2010 U.S. standard populations. All summary statistical analyses were performed in SAS version 9.4 (SAS Institute Inc., Cary, NC, USA).

### 2.4. Identification of Geographic Clusters

Circular geographic clusters of high or low MI hospitalization risks were investigated and identified during each of the 2-year time intervals using circular spatial scan statistics (CSSS) in SaTScan version 9.4.0 software (Kulldorff and Information Management Services, Inc., Boston, MA, USA) [31]. Model specifications were: (a) a discrete Poisson probability model; (b) adjustment for both age and sex as confounders; and (c) use of non-overlapping, circular, purely spatial windows. We used a maximum spatial window size of 13.4% of Florida’s population. This was chosen to ensure that identified clusters are not unusually large and that the largest county (Miami-Dade) had a chance of being part of a cluster. Likelihood ratio test (LRT) was used to assess statistical significance of potential clusters, whose p-values were generated using 999 Monte Carlo replications. We assessed statistical significance of potential clusters using a critical p-value of 0.05.

Irregularly-shaped (non-circular) spatial clusters were investigated and identified using Tango’s flexible spatial scan statistics (FSSS) in FleXScan version 3.1.2 software (National Institute of Public Health, Tokyo, Japan) [32]. These clusters would not be detected by the CSSS. Model specifications were as follows: (a) we used age- and sex-adjusted counts; (b) implemented Poisson probability model; (c) restricted likelihood ratio test (RLRT) was used to ensure that counties with non-elevated risks were not included in the high-risk cluster [33]; (d) alpha of 0.2 [34] and (e) maximum geographic cluster size of 34 counties (equivalent to approximately 50% of the number of counties in Florida).

### 2.5. Mapping of Hospitalization Risks and Clusters

All computed MI hospitalization risks and identified geographical clusters were mapped using ArcGIS Version 10.6.1 (Environmental Systems Research Institute, Redlands, CA, USA) [35]. Jenk’s optimization classification scheme was used to determine break-points for hospitalization risk maps. Only statistically significant (*p* < 0.05) high-risk clusters with relative risks (RR) ≥ 1.2 (for rural areas) and ≥ 1.1 (for urban areas) were mapped based on findings by Prates et al. [36]. Similarly, only statistically significant (*p* < 0.05) low-risk clusters, with an RR of ≤ 0.8 (for rural areas), and those with RR ≤ 0.9 (for urban areas), were mapped.

### 2.6. Temporal Trends

Temporal trends in MI hospitalization risks were investigated using plots of the annual hospitalization risks vs. time (in years) for counties with persistently high- or low-risk clusters during the study period. We calculated percentage changes in MI hospitalization risks between the time periods 2013–2014 and 2005–2006. Disparities in MI hospitalization risks were assessed by computing the risk difference between the high-risk clusters and the low-risk cluster with the lowest MI hospitalization risk, both at the beginning and at the end of the study periods.

### 2.7. Ethical Consideration

This study was approved by the University of Tennessee, Knoxville (Approval # UTK IRB-17-03601-XP) and Florida Department of Health (Protocol # 170032UTN) Institutional Review Boards (IRB) as expedited review.

### 2.8. Data Availability

All the analytic methods used to conduct the research have been described in detail for purposes of reproducing the results or replicating the procedure. However, due to the sensitive nature of the myocardial infarction hospitalization data analyzed for this study, we are unable to make the data available publicly. The data belong to a third party (Florida Agency for Health Care Administration). The authors were able to access the data through a formal Data Use Agreement with this agency. Requests to access the dataset from qualified researchers trained in human subject confidentiality protocols may be sent to the Florida Agency for Health Care Administration by contacting the Public Records Office at PublicRecordsReq@ahca.myflorida.com.

## 3. Results

### 3.1. Descriptive Analyses of MI Hospitalization

There were 428,275 inpatient principal MI hospitalization cases in Florida between 2005 and 2014. State-wide overall annual age- and sex-adjusted MI hospitalization risks as estimated using the 2010 U.S. standard were 22.0 (2005–2006), 19.8 (2007–2008), 18.4 (2009–2010), 18.0 (2011–2012), and 17.7 (2013–2014) cases/10,000 population. Those estimated using the 2000 U.S. standard population were 19.9 (2005–2006), 17.9 (2007–2008), 16.6 (2009–2010), 16.3 (2011–2012), and 15.8 (2013–2014) cases/10,000 population. Thus, MI hospitalization risks decreased by 20% overall during the 10-year study period.

Table 1 and Table 2 show state-level MI hospitalization risks adjusted to the age distributions of 2010 and 2000 U.S. standard populations, respectively, by sex, age group, race, ethnicity and rurality at the beginning and at the end of the study periods. The highest risks were observed for males, those aged 85 years or older, and non-Hispanic and rural residents, both at the beginning (2005–2006) and at the end (2013–2014) of the study periods. The risks among all groups but the “other race” category were lower by between 11.5%–26.0% during the 2013–2014 period compared to the 2005–2006 period. However, MI risks adjusted to the 2000 standard population age distributions were less than those adjusted to the 2010 U.S. standard population by 1.0–3.8 cases/10,000 persons.

### 3.2. Spatial Patterns

#### 3.2.1. Age- and Sex-Adjusted MI Risks

**Figure 1:**
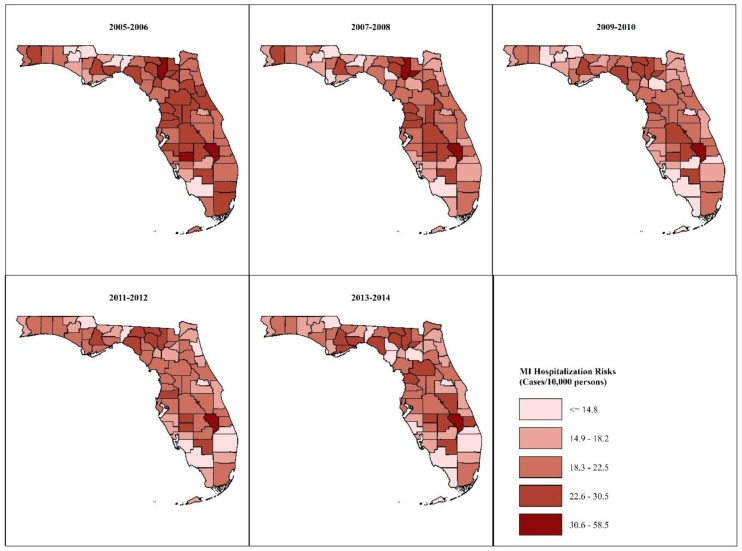
Spatial patterns of age- and sex-adjusted myocardial infarction hospitalization risks using 2010 U.S. standard population.

**Figure 2:**
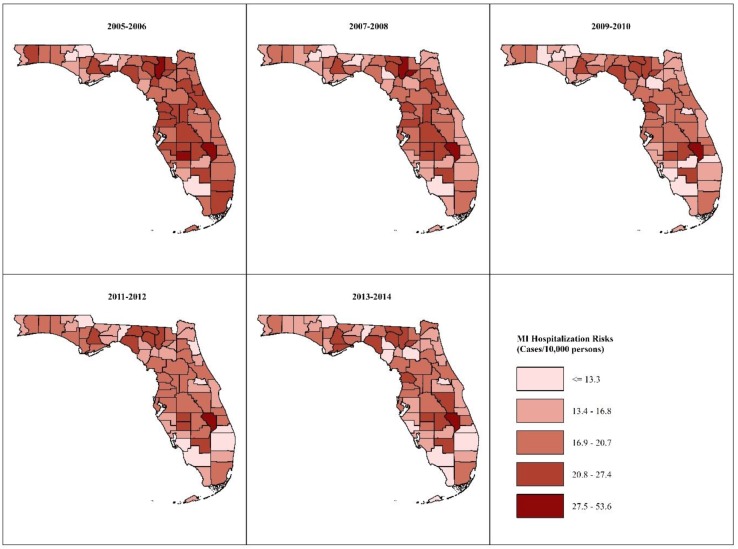
Spatial patterns of age- and sex-adjusted myocardial infarction hospitalization risks using 2000 U.S. standard population.

County-specific age- and sex-adjusted MI hospitalization risks in each of the 2-year intervals are shown in Figure 1 and Figure 2. The highest risks occurred in predominantly rural counties in the Big Bend and South Central regions of Florida, while the lowest risks occurred in mostly urban counties in southern Florida. The risks declined by between 0.5%–41.5% in most of the counties, but they increased by between 2.5% and 51% in 15 primarily rural counties scattered across the northern and middle parts of the state.

MI risks adjusted to the 2010 U.S. population census age- and sex distributions ranged from 12.0–38.7 cases/10,000 population during the 2005–2006 period to 9.6–56.4 cases/10,000 population during the 2013–2014 period. Risks adjusted to the 2000 U.S. census age- and sex distributions ranged from 10.7–34.9 cases/10,000 population at the beginning of the study to 8.8–51.4 cases/10,000 population at the end of the study. Thus, MI risks standardized to the 2010 standard population were higher by between 0.8–5.0 cases/10,000 population than those standardized to the 2000 standard population. However, the spatial patterns of MI risks appeared to be similar for both standard populations (Figure 1 and Figure 2).

#### 3.2.2. Kulldorff’s Circular Spatial Scan Statistics (CSSS) Clusters

**Figure 3:**
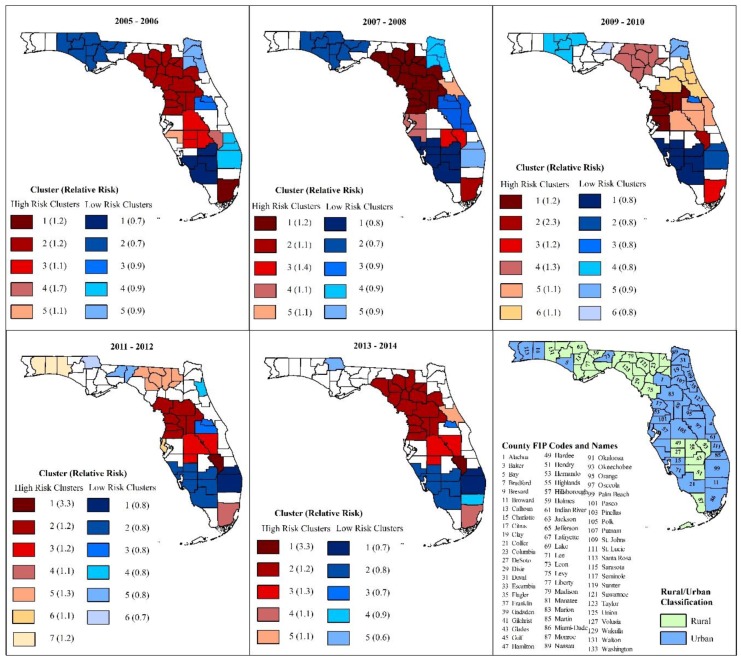
Spatial circular clusters of high and low myocardial infarction hospitalization risks and rural/urban county classification.

Figure 3 shows the geographic location of Kulldorff’s CSSS high- and low-risk MI hospitalization clusters, as well as the rural–urban designation of Florida’s counties based on Florida Department of Health Office of Rural Health definition of rural county (i.e., density of less than 100 persons per square mile) [37]. A total of 5–7 high- and 5–6 low-risk clusters were identified for each time period assessed. Consistent with the visual patterns for age- and sex-adjusted risks (Figure 1 and Figure 2), large high-risk clusters were identified in southeast Florida and the Big Bend area. Large low-risk clusters were identified in Northeast, Southeast and Southwest Florida (Figure 3). Smaller high- and low-risk clusters were located in South Central Florida and the Panhandle, respectively.

Figure 3 also shows that four low- and 3–4 high-risk clusters were observed from the beginning to the end of the study period, with 85.7% of southern Florida low-risk counties and 88% of high-risk counties in 2005–2006 retaining their status in 2013–2014. Large, persistently occurring high-risk clusters were located in Southeast Florida (13% of population) and in predominantly rural counties in the Big Bend area (11% of total population) (Table 3). Smaller persistently high-risk clusters, comprising less than 5% of the state’s population, were located in rural counties in South Central Florida. Persistently low-risk clusters comprised 5%–8% of the state’s population, and they were located in primarily coastal urban counties designated as retirement designations in Southeast and Southwest Florida (Table 4).

While some counties persisted in either low- or high-risk clusters throughout the 10-year study period, other counties experienced substantial changes in their cluster status. These changes were most evident in Northwest and Northeast Florida, where 93% of counties located in low-risk clusters in 2005–2006 transitioned to no cluster by 2013–2014. Four South Florida counties transitioned from no cluster in 2005–2006 to low-risk clusters in 2013–2014. Only two counties in Central Florida transitioned from high-risk to no cluster and vice versa. No county transitioned from high- to low-risk cluster or from low- to high-risk cluster.

The relative risks (RR) for high-risk clusters ranged from 1.1 to 3.3, and from 0.5 to 0.9 among clusters with low MI hospitalization risks.

#### 3.2.3. Tango’s Flexible Spatial Scan Statistics (FSSS) Clusters

**Figure 4:**
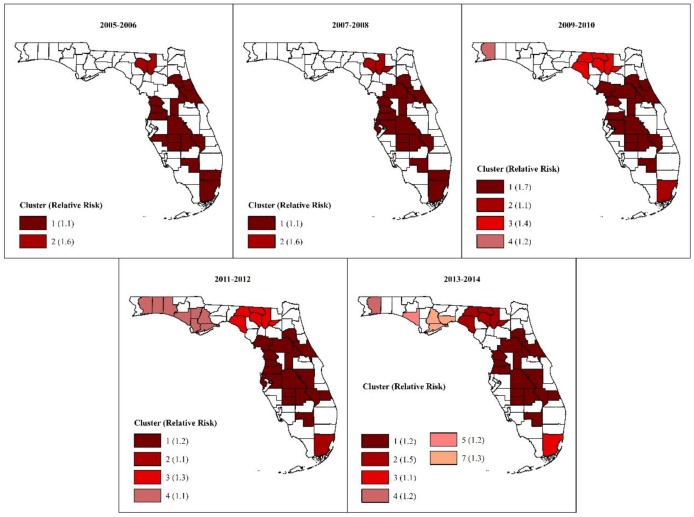
Spatial circular and non-circular clusters of high myocardial hospitalization risks.

The location and the general patterns of clustering of high MI hospitalization risks for Tango’s FSSS clusters (Figure 4) mirrored those of Kulldorff’s CSSS high-risk clusters (Figure 3). However, Tango’s FSSS clusters comprised all counties identified using Kulldorff’s CSSS, plus additional counties; hence, Tango’s clusters tended to be larger but fewer. Other notable distinctions between Kulldorff’s CSSS and Tango’s FSSS clusters include the following:

1. The identification of two high-risk circular and irregularly-shaped clusters in the Panhandle during the 2013–2014 time period. Kulldorff’s method identified a low-risk cluster in those counties during the 2005–2006 period.
2. The identification of two distinct high-risk FSSS clusters in the Big Bend area as well as persistent clustering oh high-risks in DeSoto, Hardee, Highlands, Polk and Okeechobee counties in South Central Florida throughout the 10-year study period. In contrast, Kulldorff’s method identified one large cluster in the Big Bend area in three out of five of the 2-year time intervals assessed, and persistent clustering of high risks in Polk and Okeechobee counties only.
3. The FSSS high-risk clusters only included counties with elevated risks. Kulldorff’s clusters, on the other hand, still included a few counties with elevated risks in low-risk clusters and counties with unelevated risks in high-risk clusters, despite using a window with a maximum size of 13.4% of the population in Florida. For instance, Hendry County was a part of the persistent low-risk cluster in southeast Florida despite having elevated relative risks ranging from 1.1–1.7 during the study period. Likewise, Sumter County was a constituent of the persistent high-risk cluster in the Big Bend area despite having unelevated relative risks of between 0.98–1.0 during the study period.

A comparison of FSSS clusters identified in 2005–2006 with those identified in 2013–2014 shows that 78% (14/18) of counties in high-risk clusters in 2005–2006 retained their cluster status in 2013–2014. Most of those counties were located in the middle part of the state. Thirteen counties transitioned into high-risk clusters by the 2013–2014 period, and most of those counties were located in the Panhandle.

The RR for Tango’s FSSS clusters were also lower than those for Kulldorff’s CSSS high-risk clusters (Figure 4).

### 3.3. Temporal Trends

**Figure 5:**
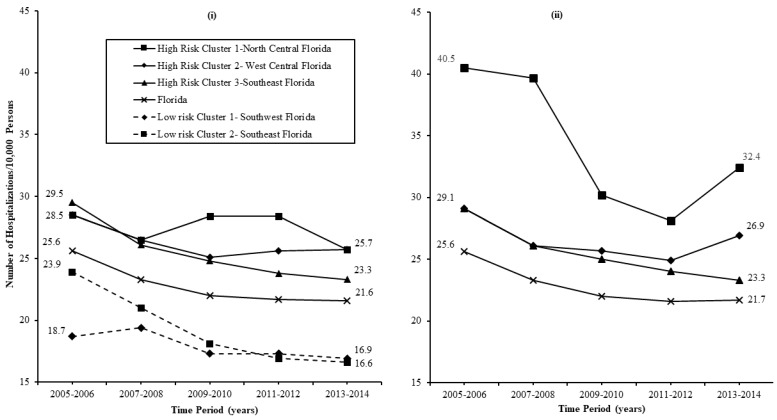
Temporal changes in myocardial infarction hospitalization risks among persistent (**i**) circular and (**ii**) circular and non-circular clusters.

The temporal trends in MI hospitalization risks among select Kulldorff’s circular and Tango’s circular and non-circular clusters that persisted over the 10-year study period are shown in Figure 5. The risks in persistent Kulldorff’s CSSS clusters declined modestly overall, declining by 9%–21% and 9%–28% in high- and low-risk clusters, respectively, between 2005–2006 and 2013–2014. Overall, we observed average rates of decline of 0.9%–2.1%/year and 0.9%–2.8% per year in high- and low-risk clusters, respectively, with clusters in southeastern Florida showing the largest declines. However, the rates of decline were not constant across the 10-year study period. Rather, the risks declined more rapidly in both high- and low-risk clusters during the first six years of study, declining by 2.2%–2.5% per year in high-risk clusters and by 1.4%–4.0% in low-risk clusters during those years. Thereafter, the rates of decline levelled off in the low-risk clusters and in the high-risk cluster in Southeast Florida, while the trajectory reversed and the risks increased by 1.2% per year in the high-risk clusters in the Big Bend region.

The risks in persistent Tango’s FSSS clusters declined by a similar magnitude (7.2%–20%) to CSSS clusters. The temporal patterns for risks in FSSS clusters were also similar to those for CSSS clusters, with risks declining more rapidly during the first 6–8 years of study, and rates of decline stalling, or the trend reversing and ticking upwards, during the latter years of study. The upward trend observed for high-risk clusters in North Central and West Central Florida during the latter years of study was more evident in FSSS clusters.

### 3.4. Health Disparities

The low-risk cluster in Southwest Florida had the lowest MI hospitalization risks (Figure 5). Therefore, MI hospitalization risks for this cluster were used as the baseline/reference for assessing changes in health disparities between circular high- and low-risk clusters during the 2013–2014 time period compared to the 2005–2006 time period.

The RD between principal MI risks in the high-risk clusters in North Central, West Central, and Southeast Florida and the referent low-risk cluster were 9.8% cases/10,000 persons, in 2005–2006, and 9.1 cases/10,000 persons in 2013–2014. This resulted in a 7.1% reduction in health disparities at the end compared to the beginning of the study periods. The RD between principal MI risks in the high-risk clusters in Southeast Florida and the referent low-risk cluster were 10.8 cases/10,000 persons in 2005–2006, and 6.4 cases/10,000 persons in 2013–2014, resulting in a 40.7% reduction in heath disparities in 2013–2014 compared to 2014.

Myocardial infarction hospitalization risks for the persistent high-risk clusters in Southeast Florida at the end of the study period (23.3 cases/10,000 persons) matched the risks for persistent low-risk clusters at the beginning of the study period (18.7–23.9 and cases/10,000 persons). However, the risks for persistent high-risk clusters in the Big Bend area and South Central Florida at the end of the study period (26.1–69.5 cases/10,000 persons) were equivalent to or greater than those for persistent low-risk clusters at the beginning of the study period (18.7–23.9 cases/10,000 persons). Thus, MI hospitalization risks for counties in high-risk clusters are at least 10 years behind those for counties in low-risk clusters.

## 4. Discussion

We used county-level state-wide data to investigate the spatial patterns and temporal trends of MI hospitalization risks at the county-level in Florida over a 10-year period to identify communities with consistently high MI burdens, so they may be prioritized for intervention to reduce health disparities.

We found modest declines in MI hospitalization risks in Florida overall and in patients stratified by age, sex, race, and ethnicity. However, there were striking geographic disparities across the state, with persistent high-risk clusters occurring in predominantly rural counties in North and South Central Florida, and persistent low-risk clusters occurring in predominantly urban counties in southern Florida. MI hospitalization risks declined modestly in all clusters, but there were disparities in the rates of decline amongst clusters, with the slowest declines occurring in high-risk clusters in northern Florida, and more rapid declines occurring in clusters in southern Florida. As a consequence, high-risk clusters in northern Florida lag behind low-risk clusters in reducing MI hospitalization risks by at least a decade and health disparities between the high and low clusters have increased. Our results can be used to inform targeted population-based primary prevention efforts for MI. Additionally, MI survivors in socioeconomically-deprived areas, such as the high-risk counties in rural Florida, are less likely to undergo coronary revascularization [38], and hence may have a higher burden of recurrent MI [19]. Therefore, our results also have implications for secondary prevention of MI.

The encouraging declines we observed in Florida overall, and in all demographic groups but the race category coded as “other”, are consistent with other studies of the temporal patterns of MI hospital admissions in disparate U.S. populations [1,2,7,9,39,40,41,42,43,44,45]. The 90.4% increase in MI hospitalization risks in the “other” race category suggests that differences in coding ethnicity data within Florida may have affected the trends we observed among racial groups.

Potential explanations for the decrease in MI hospitalization risks during the 10-year study period include changes in the sensitivity of ICD-9-CM codes for MI, increase in out-of-hospital sudden cardiac death, and a decrease in incident and recurrent MIs. However, Chen et al. [39] found concomitant declines in MI and other cardiac conditions that may be coded instead of MI, suggesting no dramatic shifts in coding hospitalizations away from MI to other cardiac conditions. Moreover, the incidence of sudden cardiac death has fallen over time, in parallel with the decline in CHD mortality [46,47], making this an unlikely explanation for the reduction in MI hospitalization risks. Furthermore, the downward trajectory occurred during a period of increased use of more sensitive troponin biomarker assays, which would be expected to increase the diagnosis of MI and MI discharges [48].

Studies conducted prior to our study showed improvements in awareness, treatment, and control of major CVD risk factors, such as low-density lipoprotein cholesterol, hypertension, and diabetes in U.S. counties [49,50,51,52,53]. A substantial increase in the utilization of interventional procedures after MI, such as Percutaneous Coronary Intervention (PCI), over the last 10 years could have also contributed to improved care of MI patients, leading to improved outcomes [42,54]. For instance, a self-organizing system based on American College of Cardiology and the American Heart Association (ACC/AHA) guidelines increased the proportion of EMS-transported ST segment elevation MI (STEMI) patients admitted directly to high volume PCI-centers in Florida from 62.4% in 2001 to 89.7% by the first half of 2009 [54]. Based on a study by De Luca et al. [55], this may have led to significantly lower reinfarctions, leading to lower hospitalization risks.

The reduction in MI hospitalization risks in our study also coincides with favorable temporal trends noted for behavioral risk factors, such as levels of sufficient physical activity and the prevalence of smoking [56,57,58,59]. Additionally, the consistency of the trend over the 10-year study period adds evidence that this is not a statistical artifact. Thus, the progressively lower MI hospitalization risks we observed in Florida over the 10-year study period likely represents a true decrease in incident and recurrent MIs [60,61,62], reflecting gains from improvements in cardiac care through primary and secondary prevention efforts [63].

Despite the overall decrease in MI hospitalization risks in Florida, the striking geographical disparities in MI hospitalization risks we observed across the state, with high-risk clusters occurring in predominantly rural counties in the Big Bend area and South Central Florida and low-risk clusters in predominantly urban counties southern Florida, suggested that health programs and efforts that contributed to the overall drop in MI discharges may have had a variable impact on different patient populations. These results corroborate prior research showing place of residence to be an important determinant of cardiovascular health [64,65].

The concentration of high-risk clusters in rural counties, coupled with the persistent clustering of high-risks in northern Florida counties, is consistent with clustering of high prevalence rates of MI hospitalizations [66], and historically high stroke and heart disease hospitalization and mortality rates in socioeconomically-deprived areas in the southeastern United States, a region that has had persistently high stroke and heart disease rates compared to the rest of the country [10,42,67,68]. This is not coincidental, since northern Florida is demographically and geographically similar to much of the southeastern United States. Moreover, the spatial patterns for MI hospitalization risks we observed in this study generally mirror the patterns of clustering previously observed for stroke, heart disease, diabetes, and hypertension rates in various county-level ecologic studies in the U.S. [10,13,14,23,69,70]. The spatial location of clusters with persistently low or high MI hospitalization risks are also remarkably similar to the location of persistent MI mortality risk clusters we identified in Florida between 2000–2014 [11]. The only notable discrepancies between MI hospitalization and mortality clusters were the persistent clustering of MI hospitalization risks in South Central Florida and the lack of persistent clustering of high MI hospitalization risks in Northwest Florida. Taken together, the concentration of high burdens of MI mortality and hospitalizations in counties previously identified as also having elevated rates of stroke, diabetes, and hypertension suggest that MI preventive and control efforts targeted to those counties would result in reductions in MI-related health disparities, as well as disparities related to stroke, diabetes, and hypertension.

The clustering of high MI hospitalization risks in rural counties likely reflects several challenges to improving cardiovascular outcomes in those counties, including financial constraints and long travel times, due to lower government spending on infrastructural resources in sparsely populated areas compared to more densely-populated areas [71]; unavailability of high-speed broadband internet services [72,73]; lack of health insurance coverage [74]; and inadequate supply of primary care providers [75], cardiologists [76], and PCI-capable hospitals [77]. Consequently, rural counties have limited capacity to implement policies and programs designed to prevent and manage CVD [78,79]. For instance, while the burden of tobacco use is higher in rural counties compared to urban counties [16], tobacco cessation programs and tobacco control policies, such as smoke-free air laws and regulations, sales tax, raising the minimum legal sales age, and restricting the advertising and sale of tobacco products, have limited geographic coverage, with rural populations receiving lower levels of protection [18,20,80]. Accordingly, the prevalence of cigarette use and other CVD risk factors is declining more quickly among high-income urban populations than low-income rural populations [6,7,58,81,82]. Rural communities also tend to have a lower prevalence of protective health-related behaviors compared to their urban counterparts [83]. Cultural attitudes towards seeking health care, lower literacy levels, higher unemployment rates, inadequate social support, and higher levels of chronic stress in rural areas may also increase risk of CVD [65,84] and attenuate the effects of efforts to improve cardiovascular health [85,86]. Variations in exposures such as extreme cold or hot temperature, air pollution, and influenza vaccination, may also have contributed to the disparities in MI hospitalization risks [87,88,89].

Potential causes for the persistent clustering of high or low MI hospitalization risks, or lower rates of decline in MI risks in rural counties in northern Florida during the 10-year study period were not investigated. However, based on similarity of the spatial patterns for MI risks with the spatial patterns for risk factors of MI, e.g., smoking [58]. hypertension [90], obesity, and lack of physical activity [57] in U.S. counties over time, persistence in MI hospitalization risks may be related to lack of temporal changes in the spatial patterns for MI risk factors. Additionally, recent economic shifts in different regions may contribute to the lag between high- and low-risk clusters [91]. Fueled by agricultural and industrial growth, tourism, retiree migration, and an expanding transportation system, southern Florida counties have undergone rapid urbanization and economic development in recent years, but North Florida has not kept pace [91,92]. Further, urban counties in southern Florida have more resources to invest in the physical and social health environment, due to higher levels of government spending in more densely populated counties. Thus, these counties may have a greater capacity to quickly adopt new models of care delivery, join campaigns for MI prevention, and implement evidence-based primary and secondary prevention strategies [93]. In contrast, counties in the more rural north tend to be chronically under resourced, which could diminish the uptake of MI interventions. Thus, cardiovascular risk has been shown to decrease in all U.S. counties, but a low-income level generates latency in this trend [82]. Not coincidentally, we observed persistent clustering of high MI hospitalization risks in counties with consistently low ranks for health factors and persistent clustering of low MI risks in counties with consistently high ranks for health factors [94]. In agreement with our study, Schieb et al. [14] found the most favorable socioeconomic and healthcare profiles for counties in persistently low-rate clusters of stroke hospitalizations, and the least favorable profiles for persistently high-rate counties. Hobbs et al. [95] reported an association of clusters of health behaviors in Queensland adults with different socio-demographic characteristics, with low-risk clusters having the healthiest profile, elevated risk-clusters having several unhealthy behaviors and moderate-risk clusters having some unhealthy behaviors. White et al. [96] described a cluster of low prevalence for hypertension, which was related to the availability of preventive primary care [84].

The unexpected high-risk cluster in Miami-Dade County may be attributed to a high prevalence of several major risk factors for MI, including hypertension (32.6%), cholesterol (32.2%) overweight/obesity (87.2%), and physical inactivity (56.7%) [97]. Moreover, Miami-Dade County has a large Hispanic and Haitian immigrant population with multiple forms of social disadvantage, including high under/uninsured rates, language barriers, and unfamiliarity with the U.S. healthcare system [98,99]. Furthermore, rates of utilization of low-cost preventive care, such as the Federally Qualified Health Centers, are quite low [99].

Since MI is a life-threatening health condition requiring immediate catherization within 90 min of first medical contact [100], MI hospitalization risk may serve as a proxy for morbidity, in which case our results showing a clustering of low MI hospitalization risks in rural counties in Northwest Florida between 2005–2010 would suggest low MI morbidity risks in Northwest Florida during that period. This contradicts the persistent clustering of high MI mortality risks we recently observed throughout a majority of northern Florida rural counties between 2000 and 2014 [11]. Therefore, the clustering of low MI hospitalization risks we observed in rural counties in Northwest Florida during the first six years of study does not imply lower MI morbidity or more favorable cardiovascular health for residents in those counties. Rather, it is indicative of higher pre-hospital MI death risks in Northwest Florida, resulting in an under-diagnosis of MI in the pre-hospital setting. Factors that may lead to underuse of cardiac care services, and hence low MI hospitalization risks, in rural counties in Northwest Florida include cultural and financial constraints, compounded by high health-uninsured rates due to limited Medicaid eligibility [101,102], scarcity of cardiac specialists [76], lack of emergency medical services to conduct lengthy patient transport on a 24 h basis [103,104], and poor availability of medical technologies such as broadband internet services [72]. Moreover, as is typical throughout the United States [105], high-volume PCI-capable hospitals are clustered in metropolitan and large urban areas on the coastline and along the major interstate highways, with 100% (*n* = 21) of rural/nonmetro counties in Florida lacking a high-volume PCI center [77]. These may result in less frequent interaction with the healthcare system, decreasing the likelihood of diagnosing MI among rural residents. Additionally, mistrust of the healthcare system, due to mistrust of the healthcare as a result of historical events such as the Tuskegee syphilis study [106], and perceived racial bias and discrimination that continues to this day, may affect healthcare-seeking behaviors and lead to an underuse of available services by minority populations [65].

Ironically, the transition of the low-risk cluster we identified in northwest Florida between 2005–2010 into a high-risk cluster between 2011–2014 may be a reflection of improvements in access to, and utilization of, cardiac care, due to mitigation of the above-mentioned barriers over time, thus reducing the risk of sudden cardiac death before hospitalization and increasing the likelihood of rural residents being hospitalized when they experience MI [107,108]. This may be attributed to concerted efforts by Florida Blue Center for Rural Health Research and Policy to improve health care access among underserved communities in rural northern Florida. Efforts of local coalitions throughout Florida have also reduced logistical barriers to timely access to PCI-based reperfusion over time, increasing the proportion of rural MI patients admitted directly to high volume PCI hospitals in Florida [54]. Additionally, increased awareness of and response to heart attack symptoms among high-risk groups [109] through educational campaigns by federal agencies, such as the CDC, and nonfederal partners, such as the American Heart Association, may have reduced pre-hospital delays in seeking timely cardiac care, thereby reducing pre-hospital MI death risks [110,111,112].

Despite the encouraging modest reductions in MI hospitalization risks, the levelling off in MI hospitalization risks in the high-risk cluster in Northwest Florida after an initial period of decline is concerning, because it suggests that the Healthy People 2020 [21] target of eliminating health disparities and improving health for all groups by 2020 may not be achieved if current trajectories continue. Moreover, the reversal of the favorable temporal trends in the high-risk cluster in North Central Florida in the latter four years of study threatens to undo the progress made in reducing the MI burden in the last decades. We observed remarkably similar temporal patterns for MI mortality risks in Florida between 2000–2014 [11].

The reasons for the temporal trends in MI hospitalization risks discussed above are not clear. However, the trends mirror the slowing of the decline in CVD risk factors that has been observed in the U.S. For instance, the management and control of hypertension in the noninstitutionalized U.S. population improved between 1999–2006, but no improvements occurred from 2007 to 2010 [51]. The percentage of U.S. adults with controlled low-density lipoprotein cholesterol increased from 45.0% in 1999–2000 to 65.3% in 2005–2006, but it decreased to 63.6% by 2009–2010 [52]. The prevalence of sufficient physical activity in U.S. counties increased from 2001 to 2009, but there was little progress between 2009 and 2011. Moreover, the increase in level of sufficient physical activity was matched by an increase in prevalence of obesity in almost all counties [57]. An increase in the prevalence of diabetes mellitus, coupled with a decrease in the prevalence of low CVD risk factor profiles may also have contributed to the unfavorable MI trends [6,7,8]. These trends in risk factor management provide circumstantial evidence that the unfavorable trends in MI hospitalizations risks in the high-risk counties in northern Florida in the latter years of study may be due to deteriorating risk factor profiles in the population. Moreover, our results showing increasing MI risks in rural counties in North Central Florida during the last four years of study are consistent with Yeh et al. [7], who showed that the growth of certain CVD risk factors, including obesity and diabetes mellitus, has disproportionately impacted certain geographic regions, particularly rural counties in Southern and Southeastern U.S. states. The great economic recession of 2008–2009 may also have resulted in higher unemployment rates in socioeconomically disadvantaged areas, further exacerbating the MI burden in rural areas in Northwest and North Central Florida. Li et al., showed an upward trend in MI occurrences in low-income, but not in high-income, populations in the Raritan Bay region, New Jersey after the onset of the 2008–2009 great recession [113]. More years of data and continued population-based surveillance of MI hospitalizations in those counties are warranted to confirm these trends. Appropriate strategies can then be implemented to prevent a reversal of many of the public health gains of the past decades.

The fact that risks for the high-risk cluster during the time period 2013–2014 were equivalent or higher than low-risk cluster risks during the 2005–2006 time period shows that high-risk clusters would need at least 10 additional years to achieve the low hospitalization risks of low-risk counties during the 2013–2014 period. Delayed declines in MI hospitalization risks in high-risk clusters in the north may be reflective of inequities in the timing of delivery, initiation, and implementation of primary and secondary prevention of MI [114].

This study has a number of strengths and limitations. Unlike most recent studies of temporal trends of MI hospitalization risks in the U.S., that are typically limited to select populations defined by age or specific socioeconomic, geographic, and racial/ethnic characteristics [1,9,39,40,44,82,115], our study included MI hospitalization data for all noninstitutionalized Florida residents; hence, our results can be generalized to nearly all patients in Florida and in other southern U.S. states with similar demographic characteristics and a similar healthcare system to Florida. Moreover, Florida’s present racial/ethnic composition, age structure, and healthcare challenges portend the demographic shifts and potential healthcare challenges anticipated for the U.S. by 2030 [116,117]. Therefore, our findings have potential implications for future healthcare system planning for cardiac care for the rest of the U.S.

We used MI hospitalization data collected before 2015 (9th Revision Clinical Modification (ICD-9-CM) because subsequent data were collected using ICD 10th Revision Clinical Modification (ICD-10-CM). While our data may not represent the “current” MI burden in Florida, restricting our study population to the period prior to 2015 ensured that any temporal changes in MI hospitalization risks would be due to changes in disease trends and not due to changes in coding practices.

The rigorous analytic methods we used in this study has helped us to get a better understanding of the disparities in the MI burden in Florida. For instance, the use of a SaTScan window size based on the county accounting for the largest population in Florida, instead of the default window size of 50% of the population of Florida, reduced the false positive rate, which would result in better targeting, hence more efficient use of scarce resources for MI prevention and control efforts. The application of restricted likelihood in the flexible spatial scan statistic model [118] resulted in the identification of both circular and irregularly shaped spatial clusters of MI hospitalization risks. Kulldorff’s circular spatial scan statistic is not appropriate for identification of irregularly shaped clusters, and yet it is currently the standard method for cluster detection and identification of spatial clusters. All high-risk clusters, regardless of their shape, would be of interest to public health practitioners, hence, the identification of non-circular clusters will reduce the false negative rate [119] and lead to improved control of MI. Thus, while we have confidence in the Kulldorff’s CSS statistic to identify the existence of specific clusters, we have less confidence that it can precisely identify the boundaries of each cluster.

This study has some limitations that suggest important areas for further research. The first limitation arises from the ecological study design. Although the county is the preferred spatial unit of analysis where public health action is being considered, the study design is prone to ecologic fallacy. Thus, interpretations of specific associations between contextual effects, such as rural residence, and MI hospitalization risks should be made with caution, recognizing that inferences based on aggregate data do not apply to comparable individual-level data [120] Additionally, geographic analysis of the MI burden at the county-level does not identify within county disparities, which can be large. Therefore, health programs could benefit from small-area studies at the ZIP code or the census tract levels. The findings from this study will be useful in guiding such studies.

It was not possible to differentiate between MI hospital admissions that represent incident cases and those that do not. Therefore, we based MI hospitalization risks on number of hospital discharges rather than patients, hence the data may include multiple admissions for the same individual (i.e., recurrent cases) or the same event (i.e., transfer cases), if he/she had more than one hospitalization. Additionally, the AHCA data do not include MI patients who did not seek care, died before hospitalization, or were hospitalized out of state, hence there is potential for selection bias.

We did not investigate the clinical, behavioral, sociodemographic, environmental, and healthcare service factors that might be associated with the spatiotemporal disparities in MI-hospitalization risks in Florida. Therefore, future studies will need to identify locally relevant determinants of the MI disparities to enable policy makers to design more effective evidence-based interventions for reducing the MI burden in the most disadvantaged regions. Moreover, investigations of the drivers of MI risks in counties within persistent low-risk clusters may help us better understand protective factors contributing to the low MI hospitalization risks in those counties.

Lastly, cluster detection by FSSS is based on a one-tailed test that detects high-risk clusters only. Therefore, it was not possible to identify low-risk circular and irregularly-shaped clusters. The FSSS needs to be improved to address this shortcoming.

## 5. Conclusions

In general, MI hospitalization risks decreased modestly across Florida over the 10-year study period. However, there are pervasive spatiotemporal disparities, with rural counties in the Big Bend area and South Central Florida having persistently higher MI hospitalization risks and urban counties in southeastern and southwestern Florida having persistently lower risks. Moreover, counties within high-risk clusters in the north lag behind those within low-risk clusters in the south by at least a decade, and there are early signs that the temporal trends have reversed in rural counties in the Big Bend area. Thus, prevention and control strategies should be targeted to high-risk counties to optimize the efficiency of interventions geared towards reducing health disparities and improving health for all Floridians.

## Figures and Tables

**Table 1:** State-level myocardial infarction hospitalization risks adjusted to the 2010 U.S. census population age distributions (2005–2006 and 2013–2014).

Characteristic	% of Total Cases		Age-Adjusted Risks/10,000 Persons (95% CI)		% Change
	2005–2006	2013–2014	2005–2006	2013–2014	
Total MI cases	92,261	84,172	22.0	17.7	19.5
Sex					
Male	60.0	61.7	29.7 (29.4, 29.9)	23.9 (23.7, 24.1)	−19.6
Female	40.0	38.3	15.7 (15.6, 15.9)	12.1 (12.0, 12.2)	−23.0
^c^ Age (years)					
0–34	0.7	0.7	0.4 (0.4, 0.5)	0.4 (0.3, 0.4)	−14.6
35–44	4.5	3.7	8.1 (7.8, 8.3)	6.5 (6.2, 6.7)	−20.2
45–54	12.7	12.9	23.3 (22.9, 23.7)	19.8 (19.4, 20.1)	−15.1
55–64	18.9	21.8	43.2 (42.5, 43.8)	36.9 (36.4, 37.4)	−14.6
65–74	22.0	24.6	68.3 (67.4, 69.3)	53.4 (52.7, 54.1)	−21.8
75–84	25.8	21.6	103.7 (102.3, 105.0)	79.6 (78.4, 80.7)	−23.2
≥85	15.4	14.7	168.7 (165.9, 171.5)	124.9 (122.7, 127.1)	−26.0
^z^ Race					
White	87.7	80.9	21.1 (22.0, 22.3)	16.5 (16.4, 16.6)	−25.5
Black	8.5	10.7	20.2 (19.7, 20.6)	17.4 (17.0, 17.8)	−13.8
All other races	2.4	7.2	21.7 (20.8, 22.6)	41.4 (403, 42.4)	+90.4
^β^ Ethnicity					
Hispanic	12.1	15.3	19.5 (19.2, 19.9)	16.8 (16.5, 17.1)	−14.1
Non-Hispanic	86.6	82.2	22.4 (22.2, 22.6)	17.4 (17.3, 17.5)	−22.4
Rural/Urban					
Rural	5.6	6.8	24.7 (24.0, 25.4)	21.9 (21.3, 22.5)	−11.5
Urban	94.4	93.2	22.1 (21.9, 22.2)	17.4 (17.3, 17.5)	−21.3

^c^ Age-specific risks; ^z^ Missing race: 2005–2006 = 1248 cases, 2013–2014 = 984 cases; ^β^ Missing ethnicity: 2005–2006 = 1248 cases, 2013–2014 = 2162 cases.

**Table 2:** State-level myocardial infarction hospitalization risks adjusted to the 2000 U.S. census population age distributions (2005–2006 and 2013–2014).

Characteristic	% of Total Cases		Age-Adjusted Risks/10,000 Persons (95% CI)		% Change
	2005–2006	2013–2014	2005–2006	2013–2014	
Total MI cases	92,261	84,172	20.0	16.0	20
Sex					
Male	60.0	61.7	26.9 (26.6, 27.1)	21.5 (21.3, 21.66)	−20.0
Female	40.0	38.3	14.4 (14.3, 14.6)	11.1 (10.9, 11.2)	−23.3
^c^ Age (years)					
0–34	0.7	0.7	0.4 (0.4, 0. 5)	0.4 (0.3, 0.4)	−14.6
35–44	4.5	3.7	8.1 (7.8, 8.3)	6.5 (6.2, 6.7)	−20.2
45–54	12.7	12.9	23.3 (22.9, 23.7)	19.8 (19.4, 20.1)	−15.1
55–64	18.9	21.8	43.2 (42.5, 43.8)	36.9 (36.4, 37.4)	−14.6
65–74	22.0	24.6	68.3 (67.4, 69.3)	53.4 (52.7, 54.1)	−21.8
75–84	25.8	21.6	103.7 (102.3, 105.0)	79.6 (78.4, 80.7)	−23.2
≥85	15.4	14.7	168.7 (165.9, 171.5)	124.9 (122.7, 127.1)	−26.0
^z^ Race					
White	87.7	80.9	20.1 (20.0, 20.3)	14.9 (14.8, 15.0)	−25.9
Black	8.5	10.7	18.3 (17.9, 18.8)	15.8 (15.5, 16.1)	−13.8
All other races	2.4	7.2	19.8 (19.0, 20.6)	37.6 (36.7, 38.6)	+89.9
^β^ Ethnicity					
Hispanic	12.1	15.3	17.7 (17.4, 18.1)	15.2 (14.9, 15.4)	−14.4
Non-Hispanic	86.6	82.2	20.4 (20.3, 20.6)	15.8 (15.7, 15.9)	−22.6
Rural/Urban					
Rural	5.6	6.8	22.4 (21.8, 23.0)	19.8 (19.2, 20.3)	−11.8
Urban	94.4	93.2	20.1 (20.0, 20.2)	15.7 (15.6, 15.9)	−21.7

^c^ Age-specific risks; ^z^ Missing race 2005–2006 = 1248 cases, 2013–2014 = 984 cases; ^β^ Missing ethnicity 2005–2006 = 1248 cases; 2013–2014 = 2162 cases.

**Table 3:** Summary statistics for circular high-risk clusters of myocardial infarction hospitalizations.

Time Interval	Cluster	County	Cluster Population (% of Florida Population)	Observed # of Hospitalizations	Expected # of Hospitalizations	# of Cases/10,000 Persons	*p*-Value
2005–2006	1	86	4,828,792 (13.4)	11,961	10,467.24	29.5	<0.00001
	2	75, 17, 41, 29, 53, 1, 83, 67, 119, 101, 121, 125, 123, 7, 23, 69, 107	3,883,180 (10.8)	13,360	11,928.25	28.5	<0.00001
	3	49, 27, 55, 105	1,427,730 (4.0)	4843	3979.55	31.1	<0.00001
	4	93	77,985 (0.2)	319	190.53	42.8	<0.00001
	5	81	620,203 (1.7)	2214	1984.07	28.5	0.00013
2007–2008	1	75, 17, 41, 29, 53, 1, 83, 67, 119, 101, 121, 125, 123, 7, 23, 69, 107	4,093,374 (11.0)	13,047	11,445.77	26.6	<0.00001
	2	86	4,931,242 (13.3)	11,042	9887.13	26.1	<0.00001
	3	55, 93	278,730 (0.8)	1266	902.22	32.7	<0.00001
	4	103, 81, 57	4,894,293 (13.2)	12,315	11,451.80	25.1	<0.00001
	5	127	997,928 (2.7)	3009	2729.53	25.7	<0.00001
2009–2010	1	93	79,951 (0.2)	392	167.89	51.4	<0.00001
	2	75, 17, 41, 29, 53, 1, 83, 67, 119, 101, 121, 125, 123, 7, 23, 69, 107	4,154,803 (11.1)	12,149	10,791.06	24.8	<0.00001
	3	105, 49, 97, 57	4,241,326 (11.3)	9068	7893.89	25.3	<0.00001
	4	86	4,982,221 (13.3)	10,704	9501.80	24.8	<0.00001
2011–2012	1	93	79,765 (0.2)	532	163.13	70.6	<0.00001
	2	17, 53, 75, 101, 119, 83, 69	3,116,403 (8.2)	10,296	8600.95	25.9	<0.00001
	3	105, 49, 97	1,828,195 (4.8)	4359	3630.45	26.0	<0.00001
	4	86	5,056,071 (13.3)	10,495	9565.79	23.8	<0.00001
	5	47, 121, 23, 79, 67, 3, 125	393,872 (1.0)	1007	776.93	28.1	<0.00001
	6	103	1,836,685 (4.8)	5225	4764.21	23.6	<0.00001
	7	91, 131, 113	790,131 (2.0)	1730	1498.07	25.0	<0.00001
2013–2014	1	93	79,952 (0.2)	532	162.99	70.7	<0.00001
	2	75, 17, 41, 29, 53, 1, 83, 67, 119, 101, 121, 125, 123, 7, 23, 69, 107	4,276,132 (11.0)	13,006	10,812.73	25.7	<0.00001
	3	105, 49, 97	1,887,107 (4.9)	4645	3729.85	27.0	<0.00001
	4	86	5,198,431 (13.4)	10,440	9840.38	23.3	<0.00001
	5	127	1,003,522 (2.6)	2818	2528.09	24.2	0.00013

**Table 4:** Summary statistics for circular low-risk clusters of myocardial infarction hospitalizations.

Time Interval	Cluster	County	Cluster Population (% of Florida Population)	Observed # of Hospitalizations	Expected # of Hospitalizations	# of Cases/10,000 Persons	*p*-Value
2005–2006	1	51, 43, 21, 71	1,853,327 (5.1)	4290	5816.98	23.9	<0.00001
	2	63, 13, 133, 59, 39, 77, 5, 131, 45, 73	1,308,614 (3.6)	1988	2690.58	18.7	<0.00001
	3	117, 95	2,977,058 (8.2)	4719	5259.91	22.9	<0.00001
	4	85, 111, 99	3,353,826 (9.3)	9590	10,269.14	23.9	<0.00001
	5	31, 89, 19, 109	2,487,636 (6.9)	4555	4987.02	23.4	<0.00001
2007–2008	1	71, 15, 51, 27, 21, 43, 115	3,085,443 (8.3)	8078	9688.30	19.5	<0.00001
	2	63, 13, 133, 59, 39, 77, 5, 131, 45, 73	1,345,777 (3.6)	1940	2582.27	17.5	<0.00001
	3	9, 97, 95, 61, 117	4,954,523 (13.3)	8728	9708.59	21.0	<0.00001
	4	31, 89, 19, 109	2,580,453 (6.9)	4141	4843.12	20.0	<0.00001
	5	99, 85	2902376 (7.8)	7241	8075.73	20.9	<0.00001
2009–2010	1	71, 15, 51, 27, 21, 43, 115	3,127,273 (8.3)	7227	9173.95	17.4	<0.00001
	2	99, 85	2,926,434 (7.8)	6304	7649.28	18.2	<0.00001
	3	117	844,417 (2.2)	1154	1492.38	17.0	<0.00001
	4	133, 59, 5, 63, 131	636,666 (1.7)	1029	1327.01	17.1	<0.00001
	5	31, 89	1,870,728 (5.0)	2890	3234.16	19.7	<0.00001
	6	73	550,260 (1.5)	668	805.64	18.3	<0.00001
2011–2012	1	99, 85	2,954,576 (7.8)	5936	7615.18	16.9	<0.00001
	2	71, 15, 51, 27, 21, 43, 115	3,173,919 (8.4)	7448	9106.19	17.7	<0.00001
	3	117, 95	3,184,374 (8.4)	4187	5050.44	18.0	<0.00001
	4	109	391,071 (1.0)	659	821.37	17.4	<0.00001
	5	65, 73	582,855 (1.5)	725	882.46	17.8	0.00002
	6	63	100,092 (0.3)	145	205.78	15.3	0.0022
2013–2014	1	99, 85	3,011,105 (7.7)	5839	7661.46	16.9	<0.00001
	2	71, 15, 51, 27, 21, 43, 115	3,270,757 (8.4)	7391	9290.84	16.5	<0.00001
	3	117	868,598 (2.2)	1163	1569.40	16.1	<0.00001
	4	11	3,582,137 (9.2)	6301	7146.82	19.1	<0.00001
	5	63	100,080 (0.3)	117	207.34	12.2	<0.00001
